# Prostate Artery Embolization Using N-Butyl Cyanoacrylate Glue for Urinary Tract Symptoms Due to Benign Prostatic Hyperplasia: A Valid Alternative to Microparticles?

**DOI:** 10.3390/jcm10143161

**Published:** 2021-07-17

**Authors:** Romaric Loffroy, Kévin Guillen, Etienne Salet, Clément Marcelin, Pierre-Olivier Comby, Marco Midulla, Nicolas Grenier, Olivier Chevallier, François Petitpierre

**Affiliations:** 1 Department of Vascular and Interventional Radiology, Image-Guided Therapy Center, François-Mitterrand University Hospital, 14 Rue Paul Gaffarel, BP 77908, 21079 Dijon, France; kguillen@hotmail.fr (K.G.); marco.midulla@chu-dijon.fr (M.M.); olivier.chevallier@chu-dijon.fr (O.C.); 2 Department of Interventional Radiology, Pellegrin University Hospital, Place Amélie-Raba-Léon, 33076 Bordeaux, France; etiennesalet@hotmail.fr (E.S.); clement.marcelin@gmail.com (C.M.); nicolas.grenier@chu-bordeaux.fr (N.G.); petitpierre.francois@gmail.com (F.P.); 3 Department of Neuroradiology and Emergency Radiology, François-Mitterrand University Hospital, 14 Rue Paul Gaffarel, BP 77908, 21079 Dijon, France; pierre-olivier.comby@chu-dijon.fr

**Keywords:** lower urinary tract symptoms, benign prostate hyperplasia, prostate artery embolization, cyanoacrylates

## Abstract

Our goal was to evaluate the feasibility, safety, and short-term outcomes of prostate artery embolization (PAE) with N-butyl cyanoacrylate (NBCA) glue as the only embolic agent in patients with benign prostatic hyperplasia (BPH)-related lower urinary tract symptoms (LUTSs). A two-center retrospective study of 50 patients (mean age, 67.6 ± 7.4 years; range, 54–85 years) treated with NBCA between 2017 and 2020 was conducted. PAE was performed using a mixture of Glubran 2 glue and Lipiodol in a 1:8 ratio, under local anesthesia, on an outpatient basis, after cone-beam computed tomography vascular mapping. Mean total injected NBCA/Lipiodol volume was 0.9 ± 0.3 mL, total injection time was 21.9 ± 7.8 s, and total radiation dose was 18,458 ± 16,397 mGy·cm. Statistically significant improvements over time occurred for the International Prostate Symptoms Score (9.9 ± 6.8 versus 20.5 ± 6.7, *p* = 0.0001), quality-of-life score (2.2 ± 1.5 versus 4.9 ± 1.0, *p* = 0.0001), prostate-specific antigen level (4.6 ± 3.0 versus 6.4 ± 3.7, *p* = 0.0001), and prostate volume (77.3 ± 30.5 versus 98.3 ± 40.2, *p* = 0.0001) at a median of 3 months versus baseline. Minor adverse events developed in 11/50 (22%) patients, but no major complications occurred. The International Index of Erectile Function did not change significantly. PAE with NBCA is feasible, safe, fast, and effective for patients with BPH-related LUTSs. Prospective comparative studies with longer follow-ups are warranted.

## 1. Introduction

Benign prostatic hyperplasia (BPH) is associated with lower urinary tract symptoms (LUTSs) and quality-of-life (QoL) alterations in aging men [1]. Transurethral resection of the prostate (TURP) remains the standard of care for most patients with failure of, or intolerance to, first-line pharmacotherapy and/or specific food supplements [1,2,3]. However, TURP can be associated with complications such as bleeding, infection, and retrograde ejaculation [2]. Furthermore, advanced age or anticoagulant therapy may contraindicate TURP. Prostate artery embolization (PAE) has become a recognized and promising therapeutic option for the management of BPH, as recent level-I evidence suggests good efficacy [4,5,6,7,8,9,10]. Despite not being recommended as first-line treatment for LUTSs due to BPH, it can be used as an alternative to standard surgical techniques, after failure to respond or intolerance to standard pharmacotherapy (α-1 adrenergic receptor antagonist and/or 5-α reductase inhibitor) [4,10].

Advantages of PAE compared to established surgical methods include performance under local anesthesia and absence of a need for postoperative physical rest [8,11]. In addition, PAE is a minimally invasive procedure that has been found safer than, and as effective as, TURP in relieving BPH-related LUTSs as measured with the International Prostate Symptom Score (IPSS) [11,12,13]. The European Association of Urology has stated that, despite the longer procedural time compared to TURP, the lesser blood loss and shorter hospitalization time support the use of PAE [14]. PAE is an advanced embolization technique usually performed with nonspherical particles (polyvinyl alcohol) to occlude the prostatic arteries [4]. No published study has compared the various commercialized embolic agents. Most studies relied on the free-flow injection of microspheres measuring 300–500 μm in diameter. Smaller particles (100–300 μm) are not recommended due to a higher risk of adverse events [4,15].

Liquid adhesive agents, notably N-butyl cyanoacrylate (NBCA), have shown excellent performance in controlling active bleeding from peripheral arteries [16,17]. NBCA is a liquid that polymerizes upon contact with ion-rich fluids such as blood. It has several advantages over other embolic agents [17,18]. Its quick administration and rapid polymerization allow fast hemostasis, which is extremely useful in patients with hemodynamic instability or massive focal bleeding. Due to its liquid consistency, NBCA allows distal embolization through a flow-directed strategy, particularly of small or tortuous arteries that are difficult to catheterize [17]. Furthermore, NBCA remains effective in patients with coagulopathy, which is common in gastrointestinal bleeding. Before administration, NBCA must be mixed with iodized oil (Lipiodol Ultra Fluid (UF); Guerbet, Aulnay-sous-Bois, France) to make the material radiopaque and to slow the NBCA polymerization rate [18]. However, NBCA is considered to be at higher risk for ischemic complications compared to other embolic agents, and its safe and effective handling requires substantial experience [18]. No published studies have evaluated NBCA for PAE in patients with BPH.

The goal of our study was to assess the feasibility, safety, and short-term efficacy of NBCA as an embolic agent for PAE in patients with symptomatic BPH.

## 2. Materials and Methods

### 2.1. Study Population

Consecutive patients treated by PAE for disabling BPH-related LUTSs at two academic centers between November 2017 and October 2020 were included in this retrospective study. Inclusion criteria were as follows: LUTSs attributed to BPH, LUTS duration ≥6 months, failure to respond or intolerance to standard pharmacotherapy (α-1 adrenergic receptor antagonist and/or 5-α reductase inhibitor) in patients contraindicated to or who refused surgery, IPSS > 7, IPSS-related QoL score > 2, and prostate volume > 40 mL. Exclusion criteria were biopsy-confirmed prostate cancer, active urinary tract infection, advanced atherosclerosis with tortuosity of the pelvic arteries, and advanced renal failure.

Our ethics committee approved the study and waived the requirement for informed patient consent in compliance with French legislation on retrospective studies of anonymized data.

### 2.2. Endovascular Procedure

All PAE procedures were performed by two experienced interventional radiologists (RL and FP) who were familiar with the procedure and followed the latest guidelines. PAE was performed on an outpatient basis, under local anesthesia, with no bladder catheter, usually via the femoral approach. An Artis Pheno (Siemens, Erlangen, Germany) or Allura XD 20 Clarity (Philips, Best, The Netherlands) angiography suite was used for 30 and 20 patients, respectively. A 5-French (Fr) sheath (Progreat; Terumo, Tokyo, Japan) was inserted into the right common femoral artery or left radial artery. The prostatic arterial supply was identified by selective internal iliac arteriography with a 4 Fr Cobra or Simmons type 2 catheter (Progreat; Terumo, Tokyo, Japan) on both sides. Superselective catheterization of the prostatic arteries was then performed coaxially with a 2.0–2.7 Fr microcatheter (Progreat; Terumo, Tokyo, Japan) and 0.014–0.018-inch hydrophilic guidewires. Three-dimensional (3D) rotational cone-beam computed tomography (CBCT) angiography was carried out routinely on both sides to obtain an arterial map, which avoided nontarget embolization due to anastomotic vessels.

**Figure 1:**
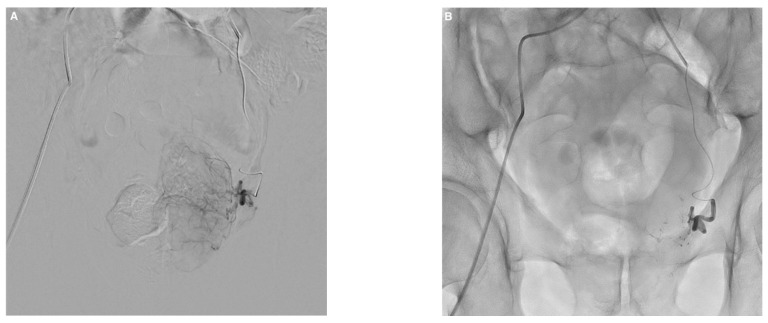

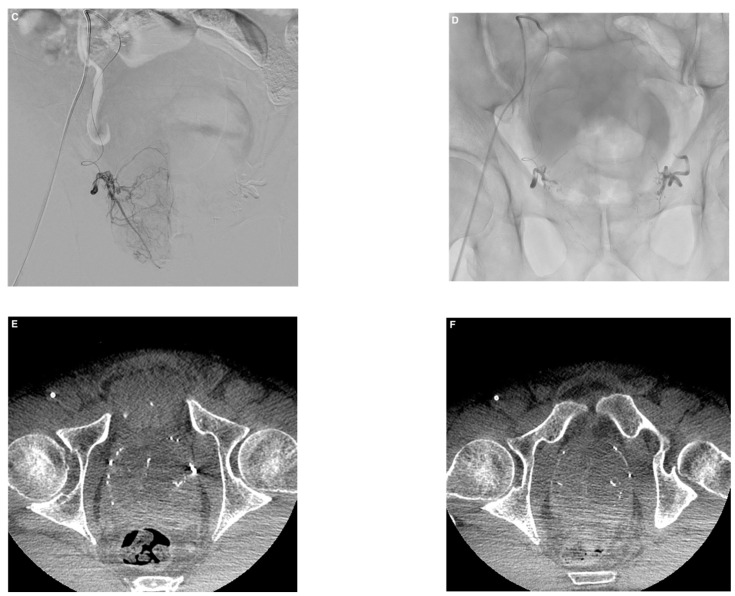
Example of prostate artery embolization (PAE) with N-butyl cyanoacrylate glue in a 74-year-old patient with symptomatic benign prostatic hyperplasia. (**A**) Left prostatic artery angiogram before PAE showing enhancement of the left prostatic lobe. (**B**) Follow-up angiogram after PAE with a mixture of Glubran^®^2/Lipiodol in a 1:8 ratio showing total occlusion. (**C**) Right prostatic artery angiogram with enhancement of the right prostatic lobe. (**D**) Visualization of the glue/lipiodol cast in the branches of the right prostatic artery after injection of the same mixture, with complete occlusion. (**E**,**F**) Axial cone-beam computed tomography images without contrast injection after bilateral PAE showing lipiodol uptake by both prostatic lobes with distal and proximal distribution of the glue/lipiodol casts. Major improvements in lower urinary tract symptoms were noted 3 months after PAE compared to baseline (IPSS, 5 versus 24; QoL score, 2 versus 6; prostate volume, 110 versus 170 mL).

Embolization was performed according to established techniques, bilaterally in most cases (Figure 1) [4]. After microcatheter positioning within the feeding artery, 2 mg of isosorbide dinitrate (Risordan, 10 mg/10 mL vial) was administered intra-arterially on each side for vasodilation. The microcatheter dead space and vascular bed of the prostatic lobe were then abundantly flushed with 10 mL of 5% dextrose solution to prevent polymerization and promote distal embolization. NBCA glue (Glubran 2, GEM; Viareggio, Italy) was diluted with iodized oil (Lipiodol Ultra Fluid; Guerbet, Aulnay-sous-Bois, France) to make the material radiopaque. A homogeneous NBCA–Lipiodol mixture was prepared immediately before the injection using two 5 mL luer-lock syringes and a three-way stopcock. A high NBCA dilution of 1:8 was used to increase mixture fluidity, thereby allowing distal embolization. Embolization was performed in free or blocked flow. Effectiveness was assessed visually during PAE, and the injection was stopped when substantial reflux occurred. The microcatheter was then promptly removed.

A vascular closure device was routinely placed at the puncture site (TR band (Terumo, Tokyo, Japan) for radial access and Exoseal (Cordis, Santa Clara, CA, USA) or FemoSeal (Terumo, Tokyo, Japan) for femoral access). Patients were monitored in the interventional unit for the first hour and in the ambulatory surgery department for 2 to 4 hours depending on whether access was radial or femoral. They were then discharged home with a prescription for 2 weeks of a prophylactic oral antibiotic and a nonsteroidal anti-inflammatory drug. Standard medical pharmacotherapy was continued for 2 weeks after PAE in all patients who were still under medication. Then, patients were asked to stop it.

### 2.3. Endpoints and Follow-Up

Data were collected before PAE (baseline) and at a scheduled follow-up visit 3 months after PAE. Clinical symptoms were assessed using the IPSS questionnaire, on which scores can range from 0 to 35 (≤7: mild symptoms; 8–19: moderate symptoms; ≥20 points: severe symptoms) and the LUTS-related IPSS-QoL score, for which responses can range from “0, delighted” to “6, terrible” [19].

The primary endpoint was the change from baseline to the 3-month visit in the IPSS score. Secondary endpoints included the International Index of Erectile Function form 5 (IIEF5, with scores ranging from “0, worst” to “25, best”), prostate volume as assessed by magnetic resonance imaging and/or ultrasound, and serum prostate-specific antigen (PSA) levels. Urodynamic testing was not performed routinely.

Technical success, defined as complete occlusion of at least one vascularizing prostate artery, and clinical success, defined as a QoL score <3, were also evaluated. Other procedural parameters such as procedure time, fluoroscopy time, radiation dose to the patient, total injected NBCA/Lipiodol mixture volume, and total NBCA/Lipiodol mixture injection time were recorded.

Last, adverse events during follow-up were reported according to the Society of Interventional Radiology (SIR) classification as minor (A and B) or major [20,21] and according to the Clavien–Dindo grading system as minor (I and II) or major (III, IV, V) [22,23].

### 2.4. Statistical Analysis

Continuous variables are described as mean ± SD (range) and qualitative/semiquantitative variables as median (interquartile range). Semiquantitative variables such as the QoL and IIEF 5 scores were handled as continuous variables given the sample size and number of modalities, after verifying the convergence of parametric and nonparametric approaches. For the IPSS score and secondary outcomes, we report only the results of the univariate analyses using the paired *t*-test. *P* values < 0.05 were considered significant. A multivariate linear regression model was built using manual backward stepwise variable selection. Several intermediate models were constructed before the optimal model was obtained. This procedure ensured the robustness of our analysis despite the exclusion of potentially interesting variables such as the center, PSA level, and number of embolized vessels. Given the small number of adverse events (all of which were minor in our study), for adjusted analyses, we dichotomized adverse events according to the SIR classification. Multivariate analysis was performed in order to find confounding factors that could have led to IPSS improvement. The statistical analyses were performed using STATA software (version 14.0, STATA, College Station, TX, USA).

## 3. Results

### 3.1. Patients

**Figure 2:**
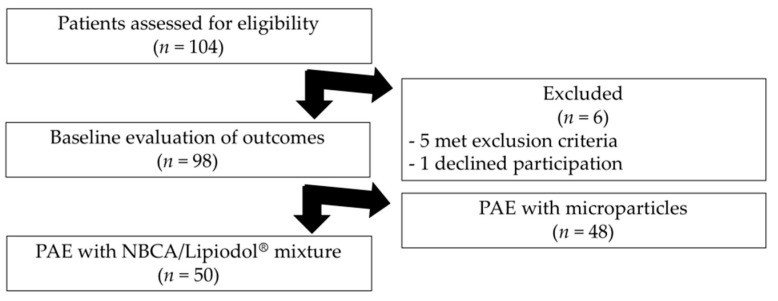
Flow chart of the study. PAE, prostate artery embolization; NBCA, N-butyl cyanoacrylate.

Figure 2 is the flow chart. Of the 104 consecutive patients assessed for eligibility, 6 were excluded and 48 were treated with microparticles, leaving 50 patients who underwent PAE with NBCA between November 2017 and October 2020 (Figure 2). Table 1 displays the baseline characteristics of the study patients.

### 3.2. Prostate Artery Embolization Procedure

Table 2 provides technical and anatomical details. Of the 50 patients, 3 (6%) required collateral embolization or protective proximal embolization of the penile artery.

Post-PAE angiograms showed complete occlusion of the targeted prostatic arteries in all 50 patients, yielding a technical success of 100%.

### 3.3. Safety and Outcomes

All patients recovered uneventfully and were discharged on the same day. No patients had major adverse events, and 11 (22%) experienced minor adverse events (Table 2). A groin hematoma developed in 5 patients and resolved spontaneously within 1–2 weeks. Transient erectile dysfunction in 4 patients abated spontaneously 2 months after PAE. One patient was diagnosed with a urinary tract infection 3 weeks after PAE and recovered with antibiotic therapy, and another had limited penile glans mucosal necrosis after PAE that healed spontaneously within about 3 weeks.

All patients were evaluated 3 months after PAE. Table 3 reports the efficacy outcomes. By univariate analysis, significant improvements vs. baseline were noted at 3 months for the IPSS, QoL score, PSA level, and prostate volume. The IIEF5 improvement was not statistically significant.

Overall and considering any clinical improvement, 45 (90%) of the 50 patients had IPSS improvement, 2 patients had stable IPSS, and 3 had IPSS worsening, after 3 months. Last, 43 (86%) of the 50 patients were satisfied after the PAE, meaning with a QoL score < 3. At 3 months, 45 (90%) of the 50 patients treated with PAE did not need pharmacotherapy anymore.

### 3.4. Prognostic Factors

By multivariate linear regression analysis, the center was not significantly associated with the IPSS or QoL score improvement at 3 months. Baseline prostate volume tended to be independently associated with the IPSS improvement, whereas no associations were found with age, anticoagulant medication, or bilateral PAE. Given the number of events and our sample size, to assess potential associations of adverse events with PAE outcomes we focused on the QoL score variation, which was not significantly different between patients with adverse events and patients without adverse events. Age, baseline IIEF5, and bilateral PAE were independently associated with the IPSS-QoL score improvement. Data are summarized in Table 4 and Table 5.

## 4. Discussion

In this retrospective study, we investigated the role of PAE with NBCA as the embolic agent for the treatment of patients with incapacitating BPH-related LUTSs. We found statistically significant improvements in the IPSS, QoL score, PSA level, and prostate volume after a median follow-up of 3 months compared to baseline. About a fifth of the patients experienced minor complications, in keeping with results obtained using microparticles [4,5,6,9,10]. No major complications were recorded. To our knowledge, this is the first clinical study reporting the use of NBCA for PAE.

The introduction of prostatic artery embolization to decrease the gland volume without surgery is a major breakthrough. Similarly, embolization of symptomatic uterine fibroids has produced comparable 5-year outcomes to those obtained with surgical excision [24]. Other studies of PAE in the same indication found similar IPSS improvements (evaluated at −10.6 points over time) with no major complications, notably no urinary incontinence or ejaculation dysfunction [9,12,13,25]. Few severe complications of PAE have been reported, with an incidence of 0.3% in a meta-analysis of 13 studies with 1254 patients [26]. None of our patients experienced major complications identified using the Clavien–Dindo or SIR classification [20,21,23]. More specifically, no patients had dysuria, acute urinary retention, hematuria, or rectorrhagia. The five puncture-site hematomas resolved spontaneously, and conservative treatment was effective in the two patients who respectively experienced a urinary tract infection and limited glans mucosa necrosis. Bilateral embolization was not associated with the IPSS improvement but seemed to influence the QoL score, although the association fell slightly short of significance. Previous data on unilateral vs. bilateral PAE are discordant [15,27]. Prostate volume was not linked to symptomatic improvement in our patients, but prostate volume decreased by 21%, in keeping with the 19% reduction in one study [9], whereas a greater reduction of 28% was obtained in another [28]. All our patients were treated on an ambulatory basis, and none required readmission for adverse events, as reported in a cohort of 486 patients [15]. Our higher technical and clinical success rates of 100% and 86%, respectively, may be partly explained by our small sample size resulting in limited power, and the corresponding proportions in a meta-analysis were 76.7% and 76.3% [26]. However, our results obtained using NBCA should be considered very promising.

The treatment of BPH-related LUTSs has changed markedly over the past 20–30 years, with a shift toward pharmacotherapy and management in primary care [1,2,3]. Until now, patients whose LUTSs become incapacitating have typically been offered TURP and, more recently, laser prostatectomy [2,8]. Compared to less invasive interventions such as urethral stenting and prostatic urethral lift, PAE may provide superior urethral patency with a comparable safety profile [14]. Although, when assessing PAE, these minimally invasive procedures seem to be more reasonable than open surgery, no such trial has been published to date [7]. Moreover, no consensus exists about the relative efficacy and safety of PAE versus TURP, although both procedures are useful and seem to have a place in clinical practice [7,29]. Compared to TURP, for at least 12 months, PAE may provide similar improvements in urologic symptom scores and quality of life. Importantly, PAE may reduce postprocedural ejaculatory dysfunction, although few data on erectile dysfunction are available [7,29]. After longer follow-ups, QoL appears similar with TURP and PAE, but the need for reintervention may be higher with PAE [30]. PAE might be a valuable alternative for the treatment of BPH-related LUTSs in selected patients in whom the symptoms are the main reason for considering surgery [7]. Knowledge of predictors is helpful to identify those patients most likely to benefit from PAE, notably with NBCA [5,6,15]. For instance, age is a crucial factor, and the risk of adverse events is significantly higher in patients older than 75 years [31].

Microparticles were historically used for PAE as well as for uterine artery embolization [12,13,24,25]. Indeed, most interventional radiologists are familiar with the use of flow-directed particulate embolization for distal devascularization. Using glue for peripheral endovascular applications such as PAE requires more experience and a deep learning curve. A large proportion of interventional radiologists are unfortunately afraid of using NBCA despite many advantages. The main advantage of using NBCA is the shorter procedural time compared to particulate embolization, which decreases the fluoroscopy time and, therefore, the radiation dose to the patient. The total mixture injection time was less than 30 s in our study, and the fluoroscopy time was generally less than 30 min. Another advantage of NBCA is that the fast polymerization from surface to core avoids the opening of pre-existing vascular anastomoses, an event reported with particles, thereby potentially decreasing the risk of nontarget embolization [12,32,33,34]. In addition, NBCA can be more efficient than other embolic agents in patients with coagulopathy, since polymerization upon contact with blood anions is not dependent on coagulation function [16,18,33,35]. Our subgroup of nearly a fifth of our population treated with anticoagulant medication did not experience smaller IPSS improvements or more adverse events [31,36]. Moreover, smaller embolic particle size is associated with a higher adverse-event rate [4,26].

NBCA/Lipiodol has several other advantages [18,35]. Lipiodol makes the embolic material radiopaque, allowing for easier fluoroscopy guidance compared with other embolic materials that are not directly visualized, such as microparticles [18]. In addition, NBCA is a liquid and can therefore be used to occlude vessels in which the microcatheter cannot be advanced, according to the blocked-flow technique. This situation is particularly frequent in PAE, as the flow rate in prostatic arteries is low. Furthermore, the lipiodol is taken up by the prostate gland, and the distribution of the treated prostate territories then becomes clearly visible by CBCT or CT. This distribution could be used as a surrogate marker of clinical success, as reported for the liver [37]. Lastly, Glubran 2 has the advantage of being cheap in Europe by comparison with microparticles (about EUR 100 per 1 mL vial versus EUR 300 per syringe) even if added Lipiodol is needed (about EUR 250 per 10 mL vial). In the end, using glue plus lipiodol is not more expensive than using microparticles.

The risk of ischemic complications after NBCA glue embolization has always been a major concern. However, our data suggest that a mixture of NBCA and Lipiodol UF may not cause a higher number of relevant ischemic complications compared to other commonly used embolic agents such as microparticles. This finding can be explained by the characteristics of NBCA [18]. The NBCA/Lipiodol ratio affects the viscosity of the liquid mixture and the NBCA polymerization rate. The ratio should be adjusted to the length of the segment to be occluded [18,32]. This adjustment provides sufficient fluidity to ensure distal embolization of the feeding artery while keeping enough viscosity to prevent excessively distal penetration into the capillary bed, thereby preserving circulation in the distal postembolic tissue via collateral channels in the intramural microcirculation [33,34]. We used a high NBCA dilution ratio of 1:8 to allow very distal embolization [38,39]. Nontarget embolization occurred in a single patient. We routinely used CBCT, which has been reported to drastically decrease the risk of nontarget embolization [40].

Precautions must be taken to minimize the complication rate. Flushing the microcatheter before the injection with dextrose to remove all ionic solutions and promptly pulling the catheter back after the injection to avoid adhesion to the vessel and trapping of NBCA are important [18,35]. Histoacryl is the most widely used NBCA glue, although it has not received the European Community (EC) mark and is not approved by the Food and Drug Administration. We used Glubran 2 (GEM Srl, Viareggio, Italy), the only EC-marked glue for endovascular administration. In Glubran 2, the comonomer methacryloxysulfolane is added to produce a more pliable and stable polymer with a lower polymerization temperature and less cytotoxicity than Histoacryl [16,18]. Thus, Glubran 2 may result in less inflammation and, therefore, in less pain. Finally, as indicated above, the use of CBCT decreases the risk of nontarget embolization.

In the present study, no comparison was performed in terms of outcomes between patients with a large intravesical median lobe and those with a nonlarge one. Maron et al. showed no significant differences in early outcomes in PAE between patients with severe (≥10 mm) and nonsevere (<10 mm) intravesical prostatic protrusion [41], meaning that selection of patients should not be focused on this specific feature. However, previous studies indicated a greater impact of PAE-induced ischemia in the adenomatous than in the stromal element of the prostate gland [42]. Most authors agree nowadays that the mechanism by which PAE resolves dynamic obstruction in patients with BPH is the shrinkage of the enlarged prostate gland as a result of PAE-induced ischemic infarction [42].

Over the last two decades, arterial embolization has gained acceptance for several indications in the urological field, either as a minimally invasive alternative to surgery or as a complementary therapeutic tool to surgery [43,44,45,46]. Indeed, arterial embolization can be used as a first-line therapy for the treatment of bleedings following partial nephrectomy, as a prophylactic therapy for large renal angiomyolipomas, or as a preoperative tool before radical nephrectomy in order to facilitate surgical intervention by decreasing intraoperative bleeding [43,44,45]. It can also be used as a palliative treatment of hemorrhage from bladder or prostate cancer [46]. The use of these minimally invasive techniques, in urological surgery as well as in interventional radiology, allows for the reduction in the risk of surgical site infection by comparison with a traditional approach. In addition, minimally invasive surgery or intervention is usually associated with better perioperative outcomes and a lower rate of overall complications [47]. Surgical or interventional site complication is one of the main risks of such approaches. In terms of wound infection, it has been shown that sutures reduce postoperative pain and improve the grade of satisfaction with the cosmetic outcome as compared to staples [48]. In interventional radiology, the systematic use of vascular closure devices can reduce puncture-site-related complications as well.

Our study has several limitations. First, the retrospective design may have led to missing data. Second, PAE with NBCA was not compared to another intervention or embolic agent. Third, although two centers contributed to patient recruitment, our sample size of only 50 patients may have limited our ability to detect significant differences and may explain some discrepancies with earlier studies. Fourth, PAE was performed by two senior interventional radiologists who had considerable experience with PAE and NBCA embolization, one in each center, resulting in a risk of bias. Fifth, the short follow-up of 3 months is a limitation, given that later recanalization has been reported after PAE with microparticles [26]. However, our goal was to report the first clinical data to date to our knowledge on PAE with NBCA to treat BPH-related LUTSs. Further studies with longer follow-ups and comparisons of embolic agents are needed. Finally, we did not have uroflowmetry or postvoid residual volume data.

## 5. Conclusions

PAE with NBCA is a feasible, safe, fast, and effective procedure with promising results in patients with BPH-related LUTSs. Further prospective comparative studies with longer follow-ups are warranted to identify the best embolic agent and the patients most likely to benefit from PAE to treat BPH-related LUTSs.

## Figures and Tables

**Table 1:** Demographics and baseline characteristics of the study population.

Characteristics	Data
Age, years	
Mean ± SD	67.6 ± 7.4
Median (IQR)	69.0 (54.0–85.0)
Urological medical history, *n* (%)	
Yes ^1^	5 (10.0)
No	45 (90.0)
Cardiovascular medical history, *n* (%)	15 (30.0)
High blood pressure/diabetes	12 (24.0)
Smoking	7 (14.00)
Dyslipidemia	5 (10.0)
Medication(s) for BPH ^2^, *n* (%)	40 (80.0)
Alpha-1 adrenergic receptor antagonists	37 (74.0)
5-alpha reductase inhibitors	5 (10.0)
Phosphodiesterase inhibitors	3 (6.0)
Others and more than one drug	17 (34.0)
Anticoagulant ± antiplatelet therapy, *n* (%)	9 (18.0)
Baseline IPSS score	
Mean ± SD	20.5 ± 6.7
Median (IQR)	20.5 (16.7–25)
Baseline QoL score	
Mean ± SD	4.9 ± 1.0
Median (IQR)	5.0 (4.0–6.0)
Baseline IIEF5 score	
Mean ± SD	16.2 ± 7.5
Median (IQR)	17.5 (11.0–23.0)
Baseline PSA, ng/mL	
Mean ± SD	6.4 ± 3.7
Median (IQR)	5.6 (4.0–7.6)
Baseline prostate volume, mL	
Mean ± SD	98.3 ± 40.2
Median (IQR)	91.2 (67.6–122.3)

IQR, interquartile range; BPH, benign prostate hyperplasia; IPSS, International Prostatic Symptoms Score; QoL, quality of life; IIEF5, International Index of Erectile Function; PSA, prostate-specific antigen. ^1^ Prostatitis (*n* = 3), obstructive pyelitis (*n* = 1), varicocele (*n* = 1). ^2^ Some patients received more than one drug.

**Table 2:** Technical features of PAE and short-term safety outcomes.

Variables	Data
Arterial approach, *n* (%)	
Left radial	11 (22.0)
Right femoral	39 (78.0)
Type of embolization, *n* (%)	
Unilateral	3 (6.0)
Bilateral	47 (94.0)
Number of embolized arteries, *n* (%)	
1	3 (6.0)
2	37 (74.0)
3	10 (20.0)
Total injected embolic mixture ^a^ volume, mL	
Mean ± SD	0.9 ± 0.3
Median (IQR)	0.8 (0.6–1.1)
Total mixture ^a^ injection time, s	
Mean ± SD	21.9 ± 7.8
Median (IQR)	20.5 (15.3–27.5)
Total PAE duration, min	
Mean ± SD	95.0 ± 29.0
Median	93 (80–120)
Fluoroscopy duration, min	
Mean ± SD	27.5 ± 11.3
Median	23.7 (19.6–33.5)
Radiation dose (mGy·cm)	
Mean ± SD	18 458 ± 16 397
Median (IQR)	14 907 (8 947–24 000)
Technical success ^b^, *n* (%)	50 (100.0)
Clinical success ^c^, *n* (%)	43 (86.0)
Complications according to SIR ^d^, *n* (%)	
Minor	11 (22.0)
A	9 (18.0)
B	2 (4.0)
Major	0 (0.0)
Clavien–Dindo score, *n* (%)	
I	9 (18.0)
II	2 (4.0)
Follow-up (months)	
Mean ± SD	4.7 ± 5.0
Median (IQR)	3.0 (3.0–5.0)

IQR, interquartile range; SIR, Society of Interventional Radiology. ^a^ Mixture of N-butyl cyanoacrylate and Lipiodol Ultra Fluid. ^b,c,d^ At median follow-up (3 months).

**Table 3:** PAE efficacy outcomes after 3 months.

Variables	Baseline	3 Months	Change (%)	*p* Value
IPSS				
Mean ± SD	20.5 ± 6.7	9.9 ± 6.8	−10.6 (51.7)	0.0001
Median (IQR)	20.5 (16.7–25)	8.0 (5.3–13.0)	−12.5 (61.0)	
QoL score				
Mean ± SD	4.9 ± 1.0	2.2 ± 1.5	−2.7 (55.1)	0.0001
Median (IQR)	5.0 (4.0–6.0)	2.0 (1.0–3.0)	−3 (60.0)	
IIEF5				
Mean ± SD	16.2 ± 7.5	15.8 ± 7.9	−0.4 (2.5)	0.078
Median (IQR)	17.5 (11.0–23.0)	18.0 (10.0–23.0)	+0.5 (2.8)	
PSA (ng/mL)				
Mean ± SD	6.4 ± 3.7	4.6 ± 3.0	−1.8 (28.1)	0.0001
Median (IQR)	5.6 (4.0–7.6)	4.1 (2.3–5.9)	−1.5 (26.8)	
Prostate volume (mL)				
Mean ± SD	98.3 ± 40.2	77.3 ± 30.5	−21 (21.4)	0.0001
Median (IQR)	91.2 (67.6–122.3)	70.7 (57.7–94.6)	−20.5 (22.5)	

IQR, interquartile range; IPSS, International Prostatic Symptoms Score; QoL, quality of life; IIEF5, International Index of Erectile Function; PSA, prostate-specific antigen. *p* values < 0.05 were considered statistically significant.

**Table 4:** Multivariate analysis: factors independently associated with the IPSS improvement at month 3 vs. baseline.

Variables	β Coefficient	95% CI	*p* Value
Age	−0.14	−0.35 to 0.03	0.167
Baseline prostate volume	0.012	−0.03 to 0.05	0.053
Bilateral PAE	2.14	−4.52 to 8.80	0.528
Anticoagulant therapy	3.70	−0.96 to 8.36	0.120

PAE, prostate artery embolization; CI, confidence interval; *β*, beta; *p* values < 0.05 were considered statistically significant.

**Table 5:** Multivariate analysis: factors independently associated with the IPSS-QoL score improvement at month 3 vs. baseline.

Variables	β Coefficient	95% CI	*p* Value
Age	−0.04	−0.07–0.00	0.024
Baseline IIEF5	−0.04	−0.07–0.00	0.049
Bilateral PAE	0.47	−0.07–0.88	0.023
Adverse events ^a^	−0.23	−0.78–0.32	0.411
Anticoagulant therapy	0.26	−0.35–0.87	0.407
Medication for BPH	−0.36	−1.05–0.32	0.297

IIEF5, International Index of Erectile Function; PAE, prostate artery embolization; *β*, beta; CI, confidence interval; BPH, benign prostate hyperplasia; ^a^ according to the Society of Interventional Radiology. *p* values < 0.05 were considered statistically significant.

## Data Availability

All the study data are reported in this article.
